# Biomarkers of DNA Damage Response Enable Flow Cytometry-Based Diagnostic to Identify Inborn DNA Repair Defects in Primary Immunodeficiencies

**DOI:** 10.1007/s10875-021-01156-7

**Published:** 2021-10-30

**Authors:** Kerstin Felgentreff, Ulrich Baumann, Christian Klemann, Catharina Schuetz, Dorothee Viemann, Martin Wetzke, Ulrich Pannicke, Sandra von Hardenberg, Bernd Auber, Klaus-Michael Debatin, Eva-Maria Jacobsen, Manfred Hoenig, Ansgar Schulz, Klaus Schwarz

**Affiliations:** 1 Department of Pediatrics and Adolescent Medicine, University Medical Center Ulm, Ulm, Germany; 2 Department of Pediatric Pulmonology, Allergy and Neonatology, Hannover Medical School, Hannover, Germany; 3 Department of Pediatrics, Medical Faculty Carl Gustav Carus, Technische Universität Dresden, Dresden, Germany; 4 Institute for Transfusion Medicine, University Ulm, Ulm, Germany; 5 Department of Human Genetics, Hannover Medical School, Hannover, Germany; 6 Institute for Clinical Transfusion Medicine and Immunogenetics Ulm, German Red Cross Blood Service Baden-Wuerttemberg - Hessen, Ulm, Germany

**Keywords:** DNA repair, DNA damage response, Immunodeficiency, Cancer susceptibility, Radiosensitivity

## Abstract

DNA damage is a constant event in every cell caused by exogenous factors such as ultraviolet and ionizing radiation (UVR/IR) and intercalating drugs, or endogenous metabolic and replicative stress. Proteins of the DNA damage response (DDR) network sense DNA lesions and induce cell cycle arrest, DNA repair, and apoptosis. Genetic defects of DDR or DNA repair proteins can be associated with immunodeficiency, bone marrow failure syndromes, and cancer susceptibility. Although various diagnostic tools are available to evaluate DNA damage, their quality to identify DNA repair deficiencies differs enormously and depends on affected pathways. In this study, we investigated the DDR biomarkers γH2AX (Ser139), p-ATM (Ser1981), and p-CHK2 (Thr68) using flow cytometry on peripheral blood cells obtained from patients with combined immunodeficiencies due to non-homologous end-joining (NHEJ) defects and ataxia telangiectasia (AT) in response to low-dose IR. Significantly reduced induction of all three markers was observed in AT patients compared to controls. However, delayed downregulation of γH2AX was found in patients with NHEJ defects. In contrast to previous reports of DDR in cellular models, these biomarkers were not sensitive enough to identify ARTEMIS deficiency with sufficient reliability. In summary, DDR biomarkers are suitable for diagnosing NHEJ defects and AT, which can be useful in neonates with abnormal TREC levels (T cell receptor excision circles) identified by newborn screening. We conclude that DDR biomarkers have benefits and some limitations depending on the underlying DNA repair deficiency.

**Supplementary Information:**

The online version contains supplementary material available at 10.1007/s10875-021-01156-7.

## Introduction

Every cell is constantly exposed to DNA damage caused by external factors such as ionizing (IR) and ultraviolet radiation (UVR), chemicals — including alkylating drugs, or by endogenous factors such as replicative and metabolic stress. While these insults may result in both DNA single-strand breaks (SSBs) and double-strand breaks (DSBs), the latter are more critical in terms of cell survival and mutation probability. Furthermore, complex lesions of base dimers and interstrand cross-links (ICL) can be induced by chemical reactions or UVR [1]. Importantly, DNA DSBs are also physiologically introduced in the T cell receptor (TCR) and immunoglobulin (Ig) genes during V(D)J recombination and class switch recombination of developing lymphocytes [2].

The cellular integrity relies on a complex network of proteins that ensure immediate sensing and efficient repair to protect the DNA from any persisting damage, known as DNA damage response (DDR). If this system fails, apoptosis, senescence, or introduction of chromosomal breaks and mutations potentially leading to neoplastic transformation are the consequences [3].

At least five DNA repair pathways — base excision repair (BER), nucleotide excision repair (NER), mismatch repair (MMR), non-homologous end-joining (NHEJ), and homologous recombination (HR) — are active through different stages of the cell cycle and used depending on the type of DNA lesion [1]. Inborn errors of proteins associated with DDR, DNA repair, and DNA stability result in various DNA repair deficiencies that can be associated with immunodeficiency, bone marrow failure, and cancer susceptibilities (Table 1).

**Table 1:**
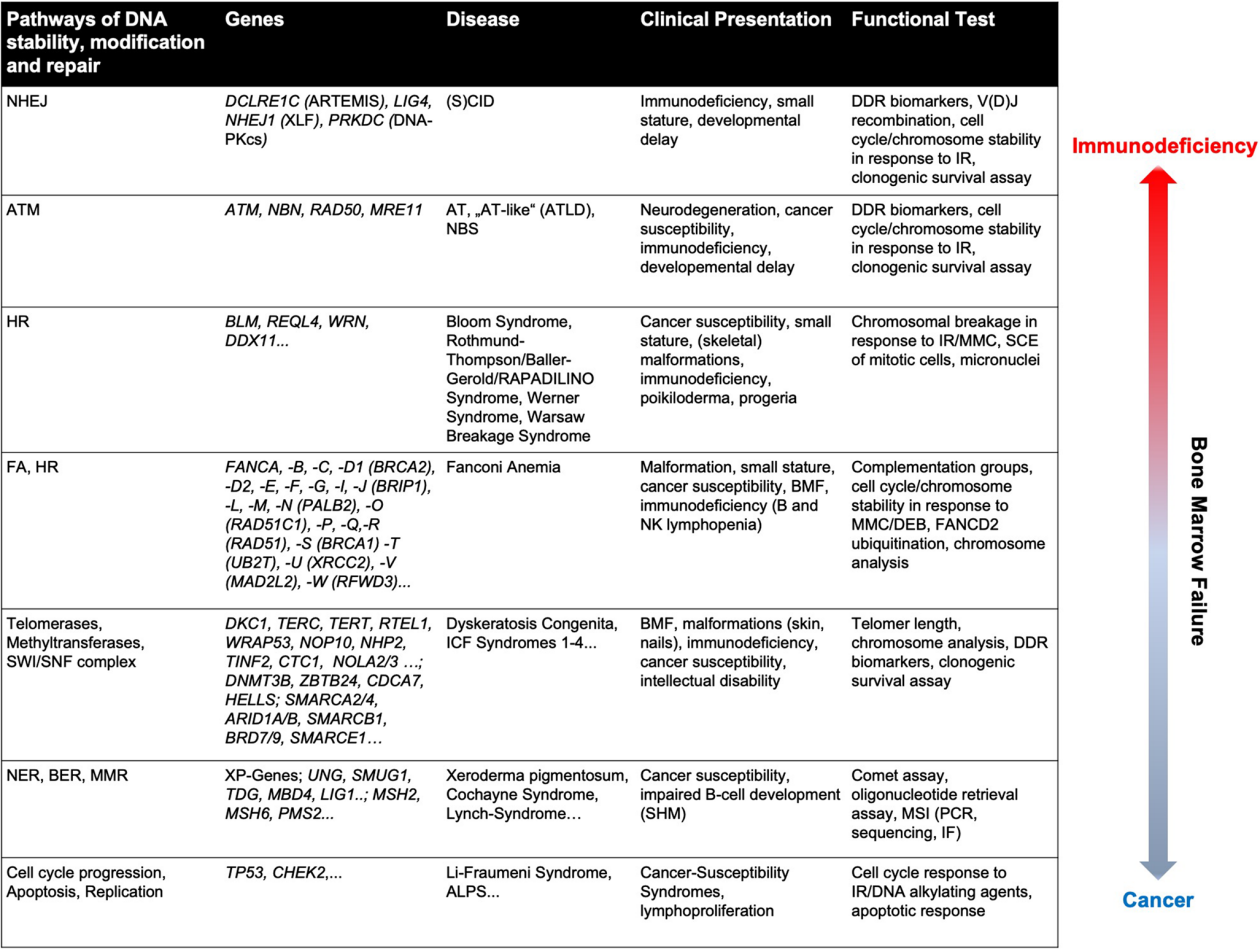
Human DNA repair deficiencies include a broad spectrum of diseases that require specific diagnostic approaches

Inborn errors of DNA stability and repair may result in immunodeficiency, bone marrow failure, and susceptibility to cancer. This table provides an overview of these rare genetic diseases, their common clinical presentation, and suitable diagnostic approaches

*SCID*, severe combined immunodeficiency; *DDR*, DNA damage response; *IR*, ionizing radiation; *MMC*, mitomycin C; *SCE*, sister chromatid exchange; *BMF*, bone marrow failure; *DEB*, diepoxybutane; *ICF*, immunodeficiency, centromere instability, facial anomalies; *SHM*, somatic hypermutation; *MSI*, microsatellite instability; *IF*, immunofluorescence; *ALPS*, autoimmune lymphoproliferative syndrome

In NHEJ-mediated repair, KU70 and KU80 are the essential sensors of free DNA ends. They bind and stabilize the DNA and further recruit the catalytic subunit of the DNA-dependent protein kinase (DNA-PKcs). Together, they form an active serine/threonine DNA-PK holoenzyme that belongs to the phosphatidylinositol 3-kinase-related kinases (PIKKs) family. This complex recruits the endonuclease ARTEMIS, which processes the DNA ends with overhangs, the XRCC4/Ligase 4 heterodimer, XLF and PAXX (paralog of XRCC4 and XLF) protein to complete the repair process [4]. Genetic defects in NHEJ-associated proteins result in severe combined immunodeficiency with a lack of T and B lymphocytes and increased cellular radiation sensitivity (RS-SCID) [5]. Most of RS-SCID patients are affected by ARTEMIS deficiency caused by genomic mutations in the *DCLRE1C* gene and present with mild radiosensitivity [6, 7]. In contrast, DNA Ligase 4 (LIG4) deficiency results in a variable phenotypic spectrum of growth failure, microcephaly, developmental delay, and a tendency to develop bone marrow failure, myelodysplasia, and/or malignancies [8–10]. Deficiency of XLF causes a rare combined immunodeficiency syndrome associated with microcephaly and developmental delay [11, 12]. So far, six patients with DNA-PKcs deficiency and variable manifestations of immunodeficiency, autoimmunity, and granulomas have been identified [13–16]. Of note, defects in XRCC4 [17–20] and PAXX [21, 22], two factors in the LIG4-ligation complex, lead to a severe DNA repair defect, but not to an immunodeficiency and are dispensable for V(D)J recombination [23].

A major sensor of DNA DSB is the MRN complex formed by MRE11, RAD50, and NBS1 that hooks free DNA ends and activates the protein kinase ATM (ataxia telangiectasia mutated) that also belongs to the PIKK family [24]. ATM is a central player in the regulation of cell cycle checkpoints, cell survival, and DNA repair [25]. Genetic defects in *ATM* lead to ataxia telangiectasia (AT) presenting with cerebellar degeneration, severe cellular sensitivity to IR, genomic instability with a predisposition to cancer [26], and antibody deficiency due to impaired class switch recombination [27]. Patients with defects in proteins of the MRN complex may present with AT-like disorder (ATLD) [28, 29], Nijmegen-Breakage Syndrome (NBS) characterized by short stature, microcephaly, “bird-like” face, mental retardation, immunodeficiency, and predisposition to develop cancers [30, 31], or NBS-like syndrome [32, 33].

Defects in the HR pathway rarely result in immunodeficiency, but rather predispose to the early development of a wide variety of cancers due to chromosomal instability. Bloom syndrome (BLM) is caused by a defect in a RecQ helicase and may present with growth deficiency, a UV-sensitive skin rash, endocrine disorders, immunodeficiency, and increased susceptibility to the development of various cancers [34]. In contrast to defects of NHEJ factors with defined overlapping function in the repair of DSB caused by V(D)J recombination, immune deficiency predominantly results from impaired development and differentiation of B lymphocytes [35].

The Fanconi Anemia (FA) complex consists of at least 22 proteins, many operating in HR, such as BRCA1, BRCA2, and RAD51 [36]. FA-proteins are needed for repair of ICLs, and induction of HR to resolve DNA DSB after ICL resection. Patients with FA can present with multiple congenital abnormalities, bone marrow failure, endocrine dysfunction, and cancer [36]. Mild immunodeficiency with impaired B and NK cell function has been reported in FA patients [37].

UV radiation-induced pyrimidine dimers are recognized and excised by proteins functioning in the NER pathway. Individuals affected by NER-deficiencies are highly sensitive to sunlight that predisposes towards benign and malignant skin tumors, and can manifest neurological abnormalities including mental retardation [38]. Defects in the BER pathway, which operates in repairing small base lesions, may lead to B cell tumors and autoimmunity, since BER is involved in mutagenic pathways of B cell development during immunoglobulin class switch recombination (CSR) and somatic hypermutation (SHM) [39]. MMR acts on the same lesions as BER and corrects errors that spontaneously occur during DNA replication, including base mismatches, short insertions, or deletions. Cells with MMR-deficiency accumulate errors leading to microsatellite instability (MSI) [40]. Besides cancer susceptibility, abnormal SHM has been reported in B cells of patients affected by MMR defects [41].

Defects of DNA telomerases and methyltransferases result in dyskeratosis congenita (DC) [42], or immunodeficiency, centromeric instability, and facial anomaly syndromes (ICF) 1–4 [43], respectively, which are associated with immunodeficiency, bone marrow failure, and cancer susceptibility. Patients affected by DC may present with a broad spectrum of combined immunodeficiency, including common variable immunodeficiency (CVID) [44, 45]. The immune defect observed in ICF syndromes is associated with impaired NHEJ and B cell development [46–48].

**Fig. 1:**
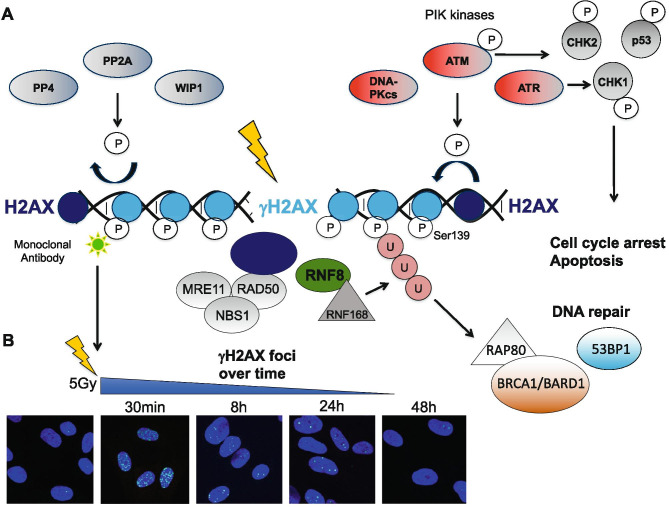
Phosphorylation of the histone protein H2AX indicates DNA DSBs. (**A**) The histone protein H2AX is phosphorylated by PIK kinases ATM, ATR, and DNA-PKcs at Ser139 leading to activation of MDC1 and recruitment of DNA repair proteins [86], (adapted and redrawn from [87]; creativecommons.org/license/by/3.0). MDC1 activates the MRN complex (MRE11, NBS1, RAD50), and E3 ubiquitin ligases RNF8 and RNF168. Ubiquitylation of H2AX results in the recruitment of various DNA repair proteins including the BRCA1/BARD1 complex and 53BP1 [86]. Once the DNA DSBs are repaired, γH2AX is dephosphorylated by WIP1, PP2A and PP4. (**B**) γH2AX foci form in nuclei of human fibroblasts in response to IR and decline after DNA DSB are repaired. Fibroblasts of a healthy donor (BJ1) were grown on cover slips, irradiated with 5 Gy and fixed at indicated time points. Immunofluorescent staining using a monoclonal antibody detecting H2AX-pSer139 and DAPI was performed to visualize γH2AX foci in the nucleus

A recent diagnostic tool to approach DNA repair deficiency is to score the activation of DDR proteins that are phosphorylated in response to DNA damage. The histone protein H2AX is phosphorylated at Ser139 (γH2AX) by PIKKs ATM, ATR, and DNA-PKcs at sites of DNA DSBs forming nuclear γH2AX foci (Fig. 1A, B). After repair of DNA lesions, γH2AX is de-phosphorylated by wild-type p53-induced phosphatase 1 (WIP1) [49], protein phosphates 2A (PP2A) [50], and PP4 [51]. Kinetics analyzed at time points between 1 and 48 h after IR can be used to evaluate DNA repair capacity. Since γH2AX formation at sides of DNA DSB was discovered, it became a popular target for the assessment of DNA damage [52]. Besides immunofluorescent staining (Fig. 1B), γH2AX foci formation can also be studied by flow cytometry in suspension cells [53].

In this study, we evaluated the activation of γH2AX (Ser139), p-ATM (Ser1981), and p-CHK2 (Thr68) in PBMCs treated with IR by flow cytometry. These markers turned out to be beneficial to detect NHEJ-defects and AT with different efficiencies. We further discuss the applicability of DDR biomarkers for diagnostic purposes and alternative approaches.

## Material and Methods

### Patient Recruitment, PBMCs Isolation, Cell Culture

Patients diagnosed with AT, and immunodeficiency syndromes, including ARTEMIS-, XLF-, DNA-PKcs-deficiencies, as well as ICF1, ICF2, and ICF4 defects, and healthy controls were recruited from University Medical Centers of Ulm, Hannover, and Dresden (Table 2). In addition, patients affected by FA, DC, and deficiencies in the MMR (*PMS2* mutation) and the SWI/SNF pathways (*SMARCA4* mutation) were included, although results are not reported. All patients and controls gave informed consent, and this study was approved by the ethic committees of Ulm University (407/16), Technical University of Dresden (TUD) (BO-EK-213052020), and Hannover Medical School (MHH) (9492-BO-K-2020). Peripheral blood mononuclear cells (PBMCs) were isolated from healthy donors (HD) and patients using Ficoll Paque Plus (GE Healthcare) according to manufacturer’s instructions. PBMCs were cultured in RPMI media supplemented with 15% fetal bovine serum (FCS) (BioWhittaker™), 1% Glutamine (Thermo Fisher Scientific), 1% non-essential amino acids (Thermo Fisher Scientific), 1% penicillin/streptavidine (Thermo Fisher Scientific), and 100 U/ml hIL-2 (R&D Systems) at 1 × 10^6/ml in a 24-well culture dish (CellStar®, Greiner Bio-One) at 37 °C 5% CO_2_ for 96 h.

**Table 2:** Summary of patients included in this study

Patient ID	Gene	Protein	Clinical presentation	Age at analysis	Biomaterial (PBMC)
ART1	*DCLRE1C*	ARTEMIS	SCID T-B-NK +	6 mo	Cryopreserved
ART2	*DCLRE1C*	ARTEMIS	SCID T-B-NK +	6 mo	Cryopreserved
ART3	*DCLRE1C*	ARTEMIS	CID T + B-NK + , severe VZV, skin abscess, vitiligo	8.5 y	Fresh
ART4	*DCLRE1C*	ARTEMIS	CID T + B-NK + , Omenn syndrome	4 mo	Cryopreserved
ART5	*DCLRE1C*	ARTEMIS	SCID T-B-NK + MFT	4 mo	Cryopreserved
DNA-PKcs1	*PRKDC*	DNA-PKcs	CID, recurrent pneumonia, alopecia areata	5.5 y	Fresh
XLF1	*NHEJ1*	CERNUNNOS/XLF	CID, agammaglobulinemia, recurrent pneumonia, chronic arthritis (adenovirus type 1), microcephaly	17.5 y	Fresh
AT1	*ATM*	ATM	B-Zell NHL	17 y	Fresh
AT2	*ATM*	ATM	Recurrent pulmonary infections, ataxia	5.5 y	Fresh
AT3	*ATM*	ATM	Identified by newborn screening	3 mo	Fresh
AT4	*ATM*	ATM	Ataxia	3.5 y	Fresh
AT5	*ATM*	ATM	Ataxia	3.5 y	Fresh
AT6	*ATM*	ATM	Ataxia, granuloma	28 y	Fresh
AT7	*ATM*	ATM	Ataxia	4.5 y	Fresh
ICF1	*DNMT3B*	DNMT3B	Chronic lung disease, hepatopathy, thrombocytopenia, anemia, lymphopenia, hypogammaglobulinemia	21 y	Cryopreserved
ICF2	*ZBTB24*	ZBTB24	Chronic lung disease, history of CMV-pneumonia, hepatopathy, metabolic disorder, hypogammaglobulinemia	3 y	Fresh
ICF4	*HELLS*	HELLS	Chronic bronchitis, mental retardation, deafness, blindness, seizures, facial dysmorphia	3.5 y	Fresh

Patients affected by inborn radiosensitive immunodeficiencies such as ARTEMIS-, DNA-PKcs-, XLF-deficiency, AT, or ICF syndromes were included in this study after giving informed consent. This table summarizes basic information of affected genes, clinical presentation, age at analysis, and sample material used

*SCID*, severe combined immunodeficiency; *CID*, combined immunodeficiency; *PBMC*, peripheral blood mononuclear cells; *VZV*, Varicella zoster virus; *MFT*, maternal–fetal T cell transfusion; *NHL*, non-Hodgkin lymphoma; *ICF*, immunodeficiency, centromere instability, facial anomalies; *CMV*, cytomegalovirus

### DNA Damage Induction, Fixation and Permeabilization

Cells were treated with 2 Gy of gamma radiation and fixed after 1 h, 4 h, 8 h, and 24 h using the solution A of Fix&Perm (Thermo Fisher Scientific) diluted 1:1 with phosphor buffered saline (PBS) (Thermo Fisher Scientific). After incubation at ambient temperature (RT) for 10 min, 2 ml chilled methanol (Roth) was added to each sample. Samples were stored at – 20 °C for at least 10 min up to 1 week.

### Surface and Intranuclear Staining for DDR Analysis

Prior fixation, PBMCs were stained with mouse anti-human CD45 Krome-Orange (Beckman Coulter), BV421 anti-human CD3 (BD Bioscience), anti-human CD56 APC (BioLegend), anti-human CD16 APC-fire (BioLegend) 1:100 in PBS supplemented with 1% FCS, and 2 mM EDTA for 30 min at RT. After permeabilization with methanol, PBMCs were washed twice with PBS/1%FCS and stained with anti-γH2AX (Ser139) FITC (clone JBW301) (Merck Millipore) 1:250, anti-p-ATM (Ser1981) PE (BioLegend) 1:200, or anti-p-CHK2 (Thr68) PE (eBioscience, Thermo Fisher Scientific) 1:50, respectively, in PBS/1%FCS for 1 h at RT. FITC Mili-Mark™ anti-mouse IgG1-k, clone MOPC-21 (Merck Millipore), and PE anti-mouse IgG1-k (BioLegend) isotype controls were used in the same concentrations as primary antibodies. Cells were washed twice, and analyzed on Navios (Beckman Coulter), or FACSAria™ (BD) flow cytometers, respectively. PBMCs obtained from AT patients were stained with surface markers after permeabilization using anti-human CD45 APC-Cy7 (BD Bioscience), anti-human CD3 APC (BioLegend) 1:100 in PBS/1%FCS for 30 min at RT, followed by staining with intranuclear markers mentioned above.

For quality control and screening of recombination capacity, non-fixed PBMCs were stained with mouse anti-human CD3 APC-Cy7 (BioLegend), anti-human CD19 FITC (BioLegend), anti-human CD56 APC (BioLegend), anti-human CD16 PE-Cy7 (BioLegend), and anti-TCRaV7.2 (BioLegend) 1:100 in PBS/1%FCS for 20 min at RT before acquisition on a FACSAria™ (BD).

### Data Analysis

Flow cytometry data were analyzed using FlowJo Vs 10.0 software. Mean fluorescent intensities (MFI) of γH2AX, p-ATM, and p-CHK2 were calculated on T and NK-cell subsets. To compare MFIs of DDR markers generated from separate experiments, fold inductions of γH2AX, p-ATM, and p-CHK2 were calculated by normalizing on MFIs of untreated samples.

Statistical analysis and generation of graphs were performed using Prism v9 software. Statistical significance was calculated by 2-way ANOVA and Tukey’s multiple comparison test. *P*-values ≤ 0.05 were considered significant (* *p* ≤ 0.05, ** *p* ≤ 0.01, *** *p* ≤ 0.001, **** *p* ≤ 0.0001).

## Results

### DDR Quantified by H2AX Phosphorylation Can Be Used for Diagnostic Purposes in Radiosensitive Immunodeficiencies

Patients diagnosed with radiosensitive combined immunodeficiencies, such as ARTEMIS-, XLF- and DNA-PKcs-deficiencies, were recruited and studied for DDR (Table 2). PBMCs were either freshly isolated from routine blood draws or obtained from cryopreserved samples. After culture for 4 days to recover from metabolic stress, PBMCs were treated with 2 Gy of IR. The γH2AX response was investigated by flow cytometry in CD45^+^CD3^+^ T and CD45^+^CD3^−^CD56^dim^CD16^+^ NK lymphocytes before IR, and 1 h, 4 h, 8 h, and 24 h after DNA damage was induced (Figure S1). Isotype controls were analyzed to define unspecific background which was subtracted from mean fluorescent intensities (MFI) of irradiated samples. Despite several limitations regarding specificity, phosphorylation of H2AX can be used as a readout for DNA DSB and the kinetics of their repair [54].

**Fig. 2:**
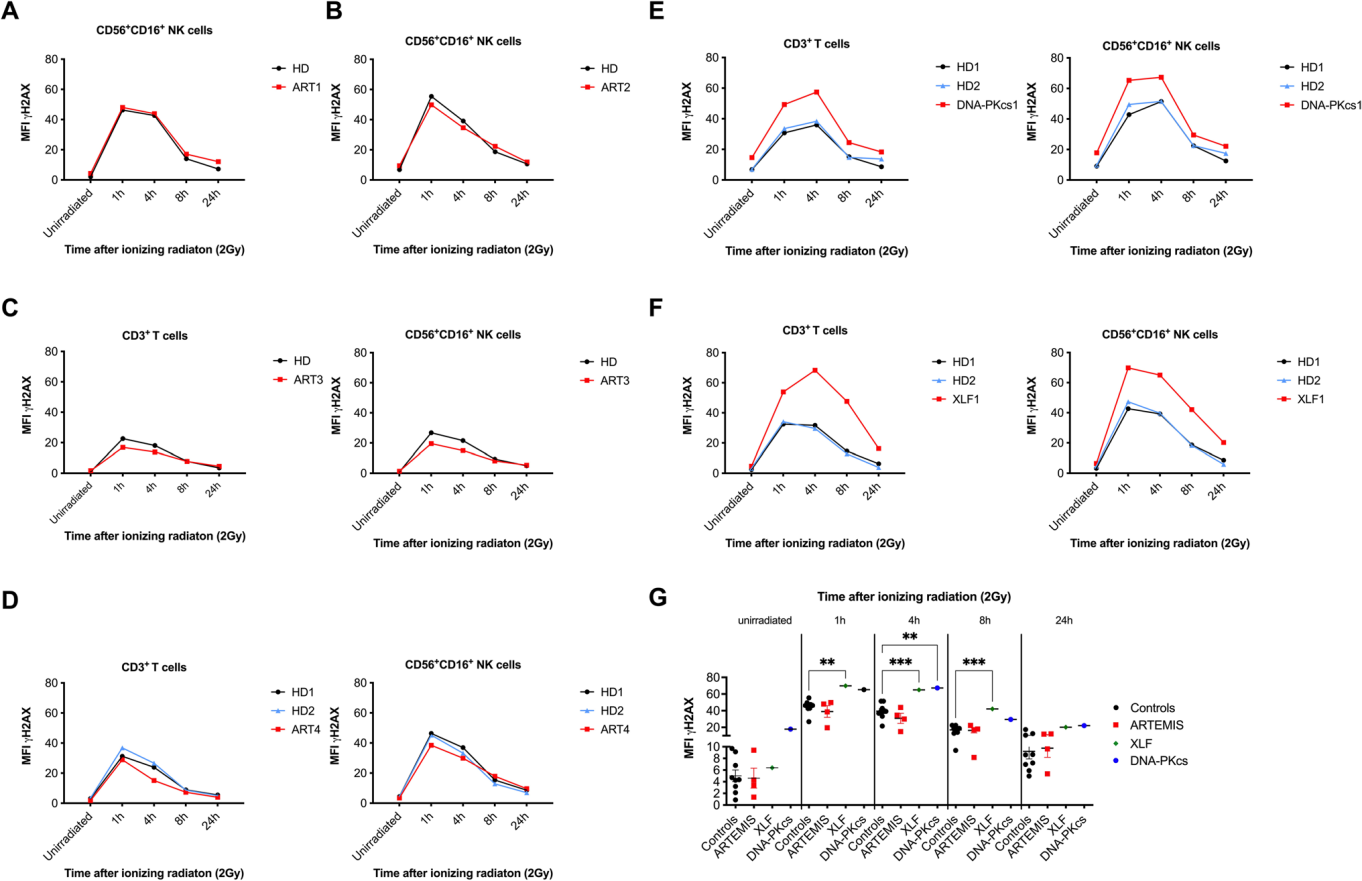
Kinetics of H2AX phosphorylation and de-phosphorylation can be used as a diagnostic tool to identify DNA repair defects in the NHEJ pathway. PBMCs obtained from 4 patients with ARTEMIS deficiency (**A**–**D**), 1 patient with DNA-PKcs deficiency (**E**), and 1 patient affected by XLF/CERNUNNOS deficiency (**F**) were irradiated with 2 Gy and fixed after 1 h, 4 h, 8 h, and 24 h. Mean fluorescence intensities (MFI) of γH2AX were analyzed by flow cytometry (Navios, Beckman Coulter) in CD45^+^CD3^−^CD56^+^CD16^+^ NK cells and CD45^+^CD3^+^ T cells, if applicable. Background quantified by appropriate isotype controls was subtracted from MFIs plotted. Healthy donors (HD) were used as day controls. MFIs of γH2AX obtained from CD45^+^CD3^−^CD56^+^CD16^+^ NK cells of healthy controls and patients with ARTEMIS, XLF, and DNA-PKcs deficiencies are shown for unirradiated samples and 1 h, 4 h, 8 h, and 24 h after IR with 2 Gy (**G**). Means are plotted as horizontal bars and statistical significance was calculated using Tukey’s multiple comparison test (* *p* ≤ 0.05, **p < 0.01, ***p < 0.001)

Besides V(D)J recombination in lymphocyte development, the endonuclease ARTEMIS is needed for end-processing of DNA breaks with overhangs that are repaired in a slower kinetic pathway [55]. Therefore, a delayed DNA repair process can be observed in patients with ARTEMIS deficiency 24–48 h after DNA damage has been induced [56, 57]. However, in contrast to investigations made on cell lines including human fibroblasts [58], we observed elevated levels of γH2AX in ARTEMIS patients compared to controls 24 h after DNA damage without significance (Fig. 2A–D, Figure S1). Of note, T cell subsets were present in two patients affected by hypomorphic ARTEMIS deficiency (ART3 and ART4). In contrast, T and NK lymphocytes from patients with other NHEJ-DNA repair deficiencies, such as DNA-PKcs and XLF deficiency, presented with elevated γH2AX levels compared to HD in response to IR (Fig. 2E, F). This could be observed at all time points and matches previous results [59]. A side-by-side comparison of mean γH2AX MFIs obtained from all healthy controls and patients revealed significant differences at 1 h, 4 h, and 8 h after IR for patients with XLF and DNA-PKcs, but not ARTEMIS deficiency (Fig. 2G).

As reported recently, genetic variants in DNA methyltransferases or helicases, resulting in ICF syndromes, also impact on NHEJ [46, 47]. We investigated three patients affected by genetic variants in *DNMT3B* (ICF1), *ZBTB24* (ICF2), and *HELLS* (ICF4) for their γH2AX-, p-ATM, and p-CHK2-mediated DDR in response to 2 Gy IR. Compared to healthy donors, increased γH2AX and p-ATM activation could be observed in patients affected by ICF1 and ICF4, however, not in the patient with ICF2 (data not shown). Very little is known about NHEJ-mediated DNA DSB repair in ICF syndromes so far. However, ICF syndromes could potentially be identified with impaired γH2AX de-phosphorylation in response to IR, and need to be differentiated from RS-(S)CIDs.

No abnormalities in DDR could be observed in PBMCs obtained from patients diagnosed with FA (*FANCA, FANCC*), DC (*RTLE1*), defects in MMR (*PMS2*), and the SWI/SNF complex (*SMARCA4*) (data not shown), although delayed reduction of γH2AX foci has been reported in FA cell lines [60].

In conclusion, γH2AX MFIs analyzed in lymphocyte subsets can be used to identify DNA repair deficiencies in the NHEJ pathway; however, the sensitivity might be too low to identify ARTEMIS defects. Although flow cytometry is faster and easier available, immunofluorescent staining of γH2AX foci in patient fibroblast lines seems to be more sensitive. Furthermore, delayed γH2AX downregulation may also be observed in patients with ICF syndromes.

### DDR Analysis as a Tool to Diagnose Ataxia Telangiectasia

**Fig. 3:**
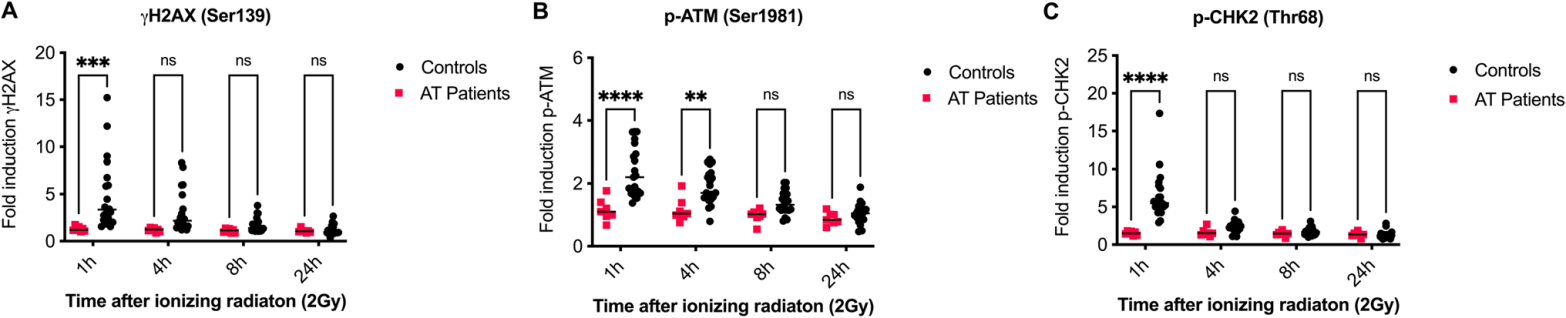
γH2AX (Ser139), p-ATM (Ser1981), and p-CHK2 (Thr68) can be used as biomarkers to identify Ataxia Telangiectasia (AT). PBMCs of seven patients affected by AT and 21 healthy controls were irradiated with 2 Gy and fixed after 1 h, 4 h, 8 h, and 24 h. Mean fluorescence intensities (MFI) of γH2AX (Ser139), p-ATM (Ser1981), and p-CHK2 (Thr68) were analyzed by flow cytometry (FACSAria™, BD) and fold inductions were calculated based on MFIs obtained from unirradiated samples. Fold inductions are shown for γH2AX (**A**), p-ATM (**B**), and p-CHK2 (**C**) in controls and patients. Means are represented by horizontal bars, statistics were calculated using two-way ANOVA and Sidaks multiple comparison test; **** *p* ≤ 0.0001, *** *p* ≤ 0.001, ** *p* ≤ 0.01, * *p* ≤ 0.05

ATM is a key factor in the DDR and a PIK kinase that phosphorylates many downstream factors including γH2AX (Ser139), p-CHK2 (Thr68), and ATM itself (Ser1981). We investigated the activation of these three DDR biomarkers in response to low dose IR with 2 Gy in CD45^+^CD3^+^ T lymphocytes from seven patients diagnosed with AT (Fig. 3). In order to compare MFIs obtained from different experiments, fold inductions of DDR biomarkers were calculated based on basal levels measured in unirradiated samples. In contrast to NHEJ-deficient lymphocytes, upregulation of DDR markers in early response to DNA damage was severely abrogated in T cells from AT patients compared to 21 healthy controls. Whereas almost no induction of γH2AX (Fig. 3A), p-ATM (Fig. 3B), and p-CHK2 (Fig. 3C) could be observed in patients, all biomarkers were activated in the controls and levels declined over time. Therefore, differences were highly significant at early time points (1 h) after IR. In particular, p-CHK2 was identified with the best selectivity, due to the highest difference among groups of patients and controls.

In summary, DDR represented by phosphorylation of H2AX, ATM, and CHK2 is a highly sensitive tool to identify patients with AT that can be readily distinguished from patients with other combined immunodeficiencies.

### Screening of V(D)J Recombination Capacity by TCRaV7.2 Expression Can Improve the Sensitivity of DDR Response

**Fig. 4:**
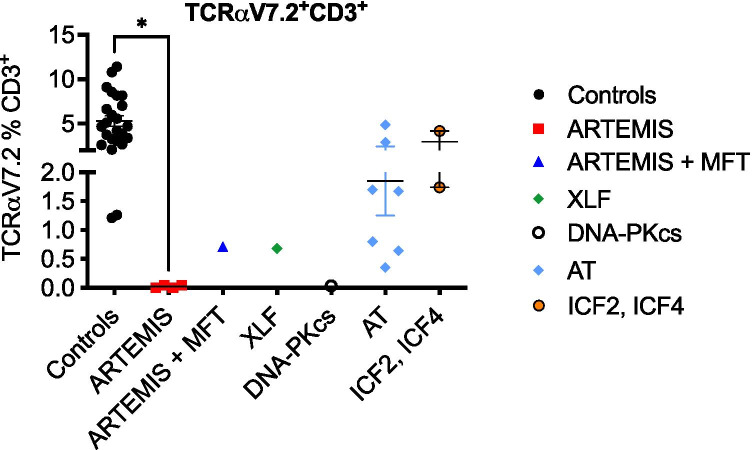
Expression of TCRaV7.2 can be used to detect NHEJ defects. Expression of the T cell receptor alpha chain V7.2 was analyzed on CD45^+^CD3^+^ T cells of five patients with ARTEMIS deficiency, including one patient with maternal–fetal T cell transfusion (MFT), one patient with DNA-PKcs deficiency, one patient with XLF deficiency, seven patients with AT, and two patients with ICF syndrome compared to 24 healthy controls. Percentages of TCRaV.2 on CD3^+^ T cells are shown. Means are represented by horizontal bars; statistics of patients versus controls were calculated using two-way ANOVA and Tukey’s multiple comparison test (* *p* ≤ 0.05)

In addition to DDR, we investigated the expression of T cell receptor alpha V7.2 (TCRaV7.2) on CD45^+^CD3^+^ T cells of all patients analyzed, including an additional patient with ARTEMIS deficiency and maternal–fetal T cell transfusion (ART5), and 21 healthy controls (Fig. 4, Figure S2). TCR-Va7.2 is a TRAV segment expressed by human T lymphocytes that represents the most distal TCRa segment and has therefore been described as a marker for recombination efficacy [61]. As reported previously, no TCRaV7.2 expression was found on T cells with ARTEMIS- or DNA-PKcs-deficiency and was reduced in XLF-deficiency and on maternal T cells of ART5 and XLF1 (Fig. 4). Of note, TCRaV7.2 expression varied among AT patients, whereas most patients were characterized by lower expression compared to controls as reported previously [61]. TCRaV7.2 expression on CD3^+^ T cells was not altered in two patients with ICF syndromes.

Although TCRaV7.2 expression might not be as specific as sequencing of *TRA* or *TRB* genes or expression analysis of various TCRVb clones, it can be used as an additional screening marker to identify reduced recombination capacity in patients with inconclusive DDR.

## Discussion

Inborn errors of DNA repair may lead to immunodeficiency, bone marrow failure, or cancer susceptibility and can be associated with growth delay, malformations, and neurological manifestations. Although often diagnosed by primary genetic testing, functional investigation still plays a role in assessing the clinical relevance. In times where whole-exome sequencing (WES) analyses are performed on a regular basis, variants of unknown significance are increasingly discovered in pathways associated with DNA stability and repair, DDR, or cell cycle and proliferation. Since DNA repair defects are associated with increased toxicity towards DNA damaging agents, including radio- or chemotherapy used for anti-cancer treatment or allogeneic hematopoietic stem cell transplantation (HSCT), a rapid diagnosis and correct classification of susceptibility is of utmost importance for outcome and survival of affected patients. Patients affected by (S)CID can be identified by newborn screening using T cell receptor excision circles (TRECs). However, also 50% of AT patients are identified with low TRECs at birth [62].

Although several diagnostic tools are available, not all of them are suitable readouts for all DNA repair deficiencies, or applicable for routine diagnostic tests. In this study, we investigated the use of flow cytometry-based DDR to identify patients affected by radiosensitive immunodeficiencies. DDR biomarkers, such as γH2AX, p-ATM and p-CHK2, were highly useful to identify patients with AT, since ATM plays a major role as a PIKK to activate these factors. Among these biomarkers, p-CHK2 showed the highest selectivity between healthy controls and affected individuals.

In contrast to AT, in which induction of DDR is impaired, NHEJ defects resulted in prolonged repair of DNA DSBs. This was characterized by elevated γH2AX levels at baseline and in response to DNA damage but did not impact on fold induction. In congruence with defects in DNA-PKcs and XLF, also LIG4-deficiency severely impairs repair of DNA DSB, which results in prolonged DDR [63, 64].

Since ARTEMIS is involved in the repair of small fractions of DNA breaks requiring end-processing [55, 65], delayed downregulation of γH2AX becomes apparent only at later time points. However, ARTEMIS deficiency could not be reliably identified by DDR analysis in PBMCs. These findings are controversial to previously reported DNA repair kinetics observed in fibroblasts [58] and Abelson virus-transformed B cell lines [56]. These discrepancies could result from differential DDR and DNA repair capacities of different cell types, which needs to be addressed in future studies. In this study, we observed increased γH2AX responses in NK compared to T lymphocytes. Whereas differential survival responses to DNA damage are well established in lymphocytes [66], DDR has not been systematically compared so far. Furthermore, immunofluorescent staining and scoring of γH2AX foci could be more sensitive than assessment by flow cytometry.

Despite recruitment from multiple centers, small cohorts of RS(S)CID patients could be investigated in this study. To verify results, larger study groups might be needed. Since DDR capacity differs among lymphocyte subsets, we recommend performing analysis on defined subsets such as T and NK lymphocytes.

In assays to quantify recombination activity, including TCRaV7.2 expression [61], NHEJ defects can be identified and separated from other diseases that might affect DDR. A combination of DDR and V(D)J recombination analysis, such as expression of TCRaV7.2, would therefore be a sufficient functional readout for defects of the NHEJ pathway, including ARTEMIS deficiency. Of note, analyses of TCRaV7.2 expression were performed on CD3^+^ T cells only, and CD161 was not included to discriminate from mucosal-associated invariant T (MAIT) cells [67].

In contrast, flow cytometry-based DDR is not suitable to confirm FA, DC, defects in BER, NER, MMR pathways, and the SWI/SNF complex, or other cancer susceptibility syndromes (Table 3). Many alternative approaches are available, also listed in Table 1. Both colony formation [68] and cellular viability assays have been gold standards to assess cellular DNA repair capacity for the last decades. This approach is highly specific for the identification of DNA repair deficiencies sensitive to IR, such as AT [69]. Alternatively, survival assays can be performed in response to DNA damaging drugs.

**Table 3:** Application of flow-cytometry based DDR analysis to diagnose DNA repair deficiencies

DNA repair deficiencies	ARTEMIS	LIG4, XLF, DNA-PKcs	AT	FA	ICF	DC	NER, BER, MMR	SWI/SNF
Suitable for flow cytometry-based DDR assay	( +) in combination with V(D)J recombination assays or TCRaV7.2*	+	+	-	( +) in combination with additional analyses of chromosome stability	-	-	-

^*^in case MFT has been ruled out

The applicability of flow cytometry-based DDR analysis as a diagnostic tool for various diseases discussed in this study is shown. Alternative approaches are shown in Table 1

*FA*, Fanconi anemia; *ICF*, immunodeficiency, centromere instability, facial anomalies; *DC*, Dyskeratosis congenita; *NER*, nucleotide excision repair; *BER*, base excision repair; *MMR*, mismatch repair; *SWI/SNF*, SWItch/sucrose non-fermentable

The comet assay is widely used for the analysis of DNA fragmentation by electrophoresis after DNA damage [70], and is an appropriate tool to study DNA repair deficiencies of multiple pathways, including NER, BER [70], AT [69], and FA [71].

Defects in DNA repair may lead to chromosomal instability represented by numerical or structural changes including amplifications, deletions, inversions, and translocations of chromosomal regions. Chromosomes can be studied in multiple ways, which is reviewed in detail by Lepage et al. [72]. Many DNA repair defects are associated with chromosomal instability [73] most commonly observed in FA, AT, Bloom syndrome, and Nijmegen breakage syndrome (NBS), but also in ATLD, ICF and NER syndromes [74], DNA ligase I (LIG1) deficiency [75], and DNA recombinase repair defects [76, 77]. Since chromosomal aberrations are rarely specific for certain diseases, chromosomal studies are rather used for monitoring purposes than for initial diagnostic. Nevertheless, structural abnormalities are highly suggestive for a DNA repair deficiency.

Mononuclear repeat microsatellite sequences are particularly sensitive to transcription errors, making them ideal targets for measuring MSI. Cells with MMR-deficiency accumulate errors, which can create new microsatellite fragments. Microsatellites can be sequenced, analyzed by fluorescent PCR, or immunofluorescent staining [78] to provide evidence for MSI.

Short telomeres are a diagnostic criterion for DC [42], but can also be found in other DNA repair deficiencies including LIG4 deficiency and Dubowitz syndrome [79].

The DDR impacts on the G1/S, intra-S, and G2/M cell cycle checkpoints that allow time for DNA repair before replication and cell division [80]. Cell cycle response to DNA damage can be a good readout for many DNA repair defects that involve proteins operating in cell cycle regulation. Failure to inhibit DNA synthesis after DNA damage (G1/S and intra-S checkpoint) is a hallmark of ATM-deficient cells [81]. A prolonged cell cycle arrest at the G2/M checkpoint can be observed in NHEJ-deficient cells [82]. Treatment with alkylating nitrogen mustard (NM) or mitomycin C (MMC) results in cell cycle arrest at G2/M in cells with defects in the FA pathway, which is frequently used to diagnose FA [83].

Furthermore, additional DDR biomarkers altered by phosphorylation can be used to study DDR and DNA repair capacity. Impaired phosphorylation of structural maintenance of chromosome 1 (SMC1) has been reported in patients with AT and was also reduced in heterozygous carriers of ATM mutations [84]. Rosen et al. reported the use of p-DNA-PKcs, p-53BP1, p-RPA2/32, p-BRCA1, p-p53, and p21 as additional DDR biomarkers in ATM-, BRCA1-, and BRCA2-deficient cell lines treated with etoposide [85].

Compared to available diagnostic tests, flow cytometry-based assays can be performed on peripheral blood cells and provide quick results. In contrast to cell cycle and cell survival assays, DDR is not dependent on proliferative response to mitogen stimulation, which can be a serious limitation in cellular material obtained from patients with primary immunodeficiencies. However, more biomarkers need to be identified to cover the heterogeneity of DNA repair deficiencies. Of note, DDR is not affected in HR- or FA-related defects, although DNA repair kinetics may be altered.

Genetic defects affecting DNA stability and repair can lead to overlapping presentations of immunodeficiency, bone marrow failure, and cancer susceptibility, and functional diagnostics can be challenging. Identification of diagnostic algorithms with the use of specific analysis tools can help to discriminate affected pathways; however, up to now, one test alone will not be able to cover the complete spectrum.

## Supplementary Information

Below is the link to the electronic supplementary material.

Figure S1: Gating strategy to calculate fluorescence intensities of γH2AX. The gating strategy is shown for a healthy control (top) and patient ART1 (bottom), both samples were obtained from cryopreserved material. After gating out doublets, we gated on CD45+ lymphocytes, and subsequently on CD3+ T cells, or CD3-CD56+CD16+ NK cells. Fluorescence intensities of γH2AX were analyzed on CD45+CD3+ T lymphocytes, if applicable (not shown in this figure), and CD45+CD3-CD56+CD16+ NK lymphocytes. Fluorescence intensities of isotype controls and γH2AX in unirradiated NK lymphocytes, and 1h, 4h, 24h after IR with 2Gy are shown in colors indicated by the legend on the right-hand side. (JPG 315 kb)

Figure S2: Gating strategy of surface antibodies detected on PBMCs of patients with NHEJ defects and healthy donors. PBMCs were isolated from healthy donors (HD) and patients with NHEJ defects described in Table 2. CD3 and CD19 expression was analyzed on lymphocytes identified by size and granularity. CD56+CD16+ NK cells were identified on CD3- lymphocytes. Expression on TCRα7.2 was analyzed on CD3+ T lymphocytes. (PDF 5416 kb)

## Data Availability

All data generated or analyzed during this study are included in this published article and its supplementary information files.
