# Supplementary figures and images for: Biomarkers of DNA Damage Response Enable Flow Cytometry-Based Diagnostic to Identify Inborn DNA Repair Defects in Primary Immunodeficiencies
Source: J Clin Immunol. 2021 Oct 30;42(2):286–98. doi: 10.1007/s10875-021-01156-7 (PMC8821069; doi:10.1007/s10875-021-01156-7)

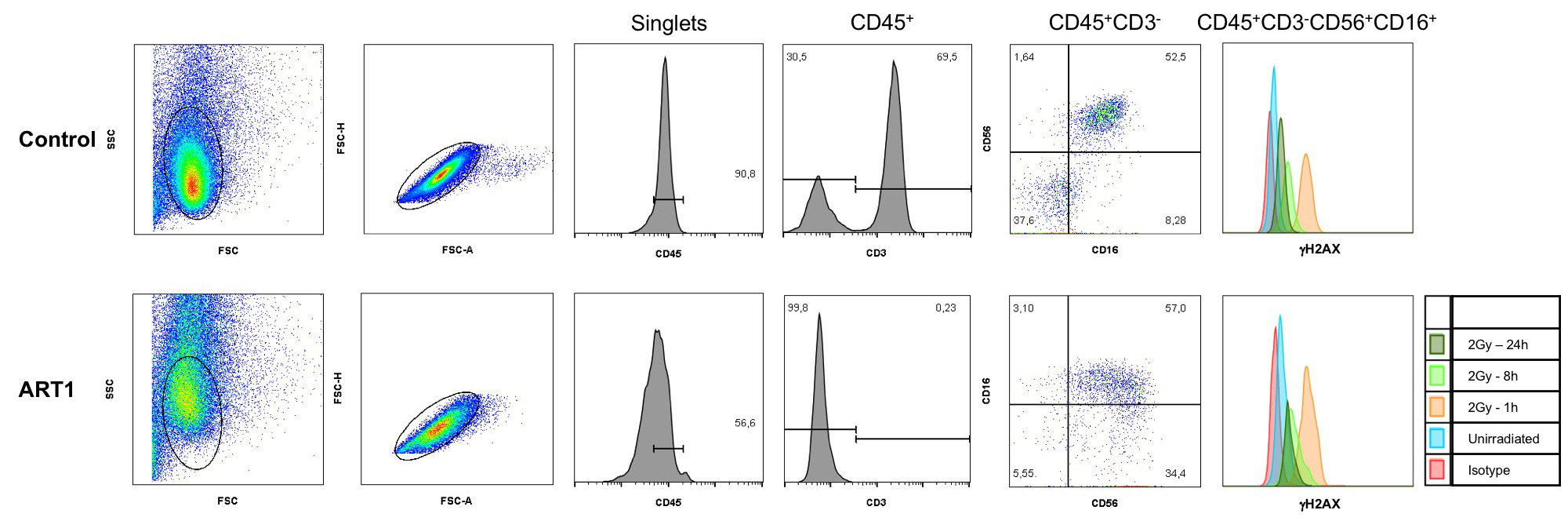

Supplement: Supplementary file 1 — Figure S1: Gating strategy to calculate fluorescence intensities of γH2AX. The gating strategy is shown for a healthy control (top) and patient ART1 (bottom), both samples were obtained from cryopreserved material. After gating out doublets, we gated on CD45+ lymphocytes, and subsequently on CD3+ T cells, or CD3-CD56+CD16+ NK cells. Fluorescence intensities of γH2AX were analyzed on CD45+CD3+ T lymphocytes, if applicable (not shown in this figure), and CD45+CD3-CD56+CD16+ NK lymphocytes. Fluorescence intensities of isotype controls and γH2AX in unirradiated NK lymphocytes, and 1h, 4h, 24h after IR with 2Gy are shown in colors indicated by the legend on the right-hand side. (JPG 315 kb) [file 10875_2021_1156_MOESM1_ESM.jpg]

HD1

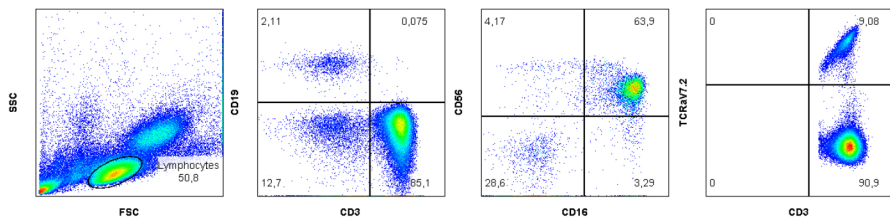

HD2

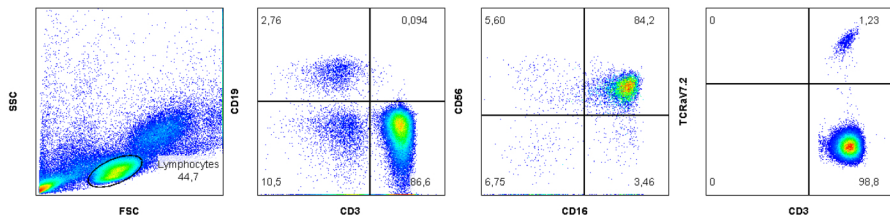

ART3

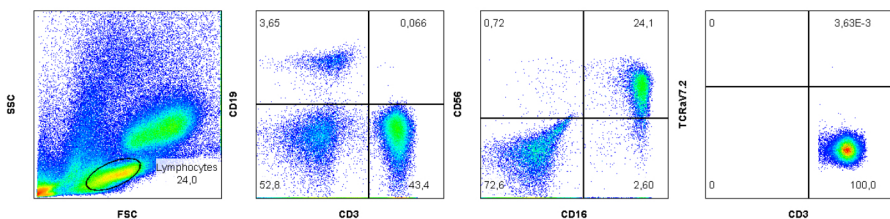

ART4

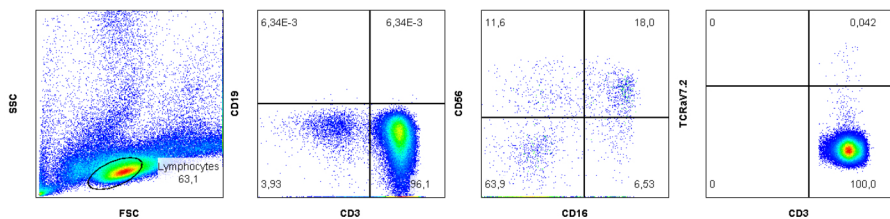

DNA-PKcs1

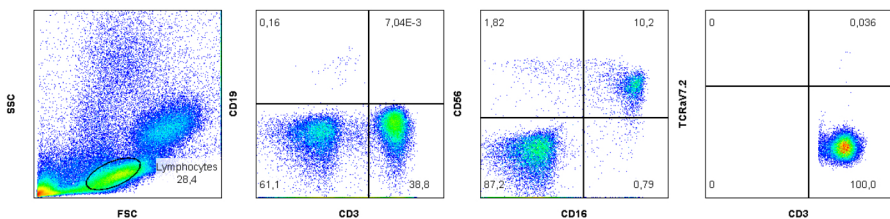

XLF1

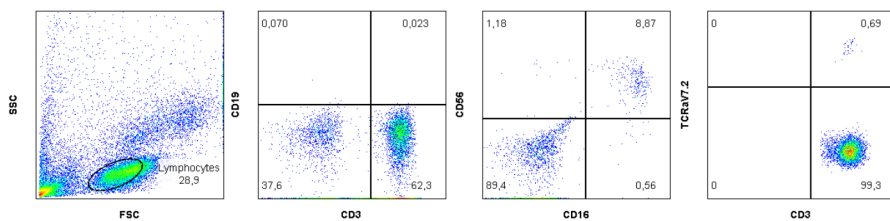

Supplement: Supplementary file 2 — Figure S2: Gating strategy of surface antibodies detected on PBMCs of patients with NHEJ defects and healthy donors. PBMCs were isolated from healthy donors (HD) and patients with NHEJ defects described in Table 2. CD3 and CD19 expression was analyzed on lymphocytes identified by size and granularity. CD56+CD16+ NK cells were identified on CD3- lymphocytes. Expression on TCRα7.2 was analyzed on CD3+ T lymphocytes. (PDF 5416 kb) [file 10875_2021_1156_MOESM2_ESM.pdf]
